# An isogenic neurovascular unit model comprised of human induced pluripotent stem cell-derived brain microvascular endothelial cells, pericytes, astrocytes, and neurons

**DOI:** 10.1186/s12987-019-0145-6

**Published:** 2019-08-07

**Authors:** Scott G. Canfield, Matthew J. Stebbins, Madeline G. Faubion, Benjamin D. Gastfriend, Sean P. Palecek, Eric V. Shusta

**Affiliations:** 1 Department of Chemical and Biological Engineering, University of Wisconsin, Madison, WI 53706 USA; 2 Present Address: Department of Cellular and Integrative Physiology, Indiana University School of Medicine, 620 Chestnut Street, Terre Haute, IN 47809 USA

**Keywords:** Blood–brain barrier, Isogenic model, Human induced pluripotent stem cells, Neurovascular unit

## Abstract

**Background:**

Brain microvascular endothelial cells (BMECs) astrocytes, neurons, and pericytes form the neurovascular unit (NVU). Interactions with NVU cells endow BMECs with extremely tight barriers via the expression of tight junction proteins, a host of active efflux and nutrient transporters, and reduced transcellular transport. To recreate the BMEC-enhancing functions of NVU cells, we combined BMECs, astrocytes, neurons, and brain pericyte-like cells.

**Methods:**

BMECs, neurons, astrocytes, and brain like pericytes were differentiated from human induced pluripotent stem cells (iPSCs) and placed in a Transwell-type NVU model. BMECs were placed in co-culture with neurons, astrocytes, and/or pericytes alone or in varying combinations and critical barrier properties were monitored.

**Results:**

Co-culture with pericytes followed by a mixture of neurons and astrocytes (1:3) induced the greatest barrier tightening in BMECs, supported by a significant increase in junctional localization of occludin. BMECs also expressed active P-glycoprotein (PGP) efflux transporters under baseline BMEC monoculture conditions and continued to express baseline active PGP efflux transporters regardless of co-culture conditions. Finally, brain-like pericyte co-culture significantly reduced the rate of non-specific transcytosis across BMECs.

**Conclusions:**

Importantly, each cell type in the NVU model was differentiated from the same donor iPSC source, yielding an isogenic model that could prove enabling for enhanced personalized modeling of the NVU in human health and disease.

## Introduction

The blood–brain barrier (BBB) is both a passive and active barrier between the CNS and its surrounding vasculature [1]. The BBB is critical in hindering the movement of pathogens and toxins from the blood to the brain, while simultaneously allowing the passage of essential nutrients [2]. Brain microvascular endothelial cells (BMECs) possess specialized features such as tight junctions, efflux transporter activity and reduced nonspecific transcytosis compared to non-brain endothelium. BMECs gain their specialized BBB functions through interactions with supporting cells such as pericytes, astrocytes and neurons that along with BMECs form the so-called neurovascular unit (NVU). The interaction and cross-talk between BMECs and the cells of the NVU is vital in the development, regulation, and maintenance of the BBB [2–8].

A number of in vitro models have been utilized to understand the role of the BBB in both physiological and pathological states [9–12]. The majority of BBB models have been based on freshly isolated BMECs from a number of different species, although interpretation of results need to be carefully weighted due to inter-species variations [13–15]. Use of human primary or immortalized BMECs addresses some of the limitations of animal-sourced BMECs, however issues with de-differentiation and suboptimal phenotypes can limit their utility [16–20]. Human induced pluripotent stem cell (iPSC)-derived BMECs have recently become available and exhibit multiple critical phenotypes: elevated TEER, reduced permeability, and the expression of tight junction proteins, nutrient transporters and efflux transporters [21–26]. Additionally, iPSC-derived BMECs and their supporting NVU cell types have been utilized in several human disease models, and could be especially powerful in modeling human genetic diseases [11, 27].

A number of approaches have been employed to further enhance iPSC-based in vitro BBB models, including small molecule addition, shear stress (flow), tissue engineering approaches, and the co-culture with other NVU cell types [23, 28–30]. Astrocyte, neuron and pericyte co-culture with BMECs individually have been shown to enhance several BBB properties, including elevated TEER, reduced para-cellular permeability and continuous tight junction formation [23, 31]. Enhancement of BMEC properties was observed when BMECs were placed in co-culture with a multicellular combination of primary pericytes, and neural progenitor-derived astrocytes and neurons [21, 25, 26, 31–34]. Moreover, the addition of retinoic acid to the iPSC-derived BMECs significantly elevated barrier tightness in conjunction with primary brain pericytes and neural progenitor-derived astrocytes and neurons, approaching in vivo TEER measurements [23]. Recently, we reported an NVU model consisting of iPSC-derived neurons and astrocytes in co-culture with iPSC-derived BMECs that were all differentiated from the same parental iPSC line [25]. Such an isogenic model could facilitate the study of BBB function in human health and disease when using patient derived iPSC lines. However, a limitation of the iPSC-derived isogenic NVU model was the lack of stem cell-derived brain pericyte influences [25, 35]. We recently described the differentiation of brain-like pericytes from human iPSCs via a neural crest lineage, and demonstrated their capacity to induce barrier properties and reduced nonspecific transcytosis in iPSC-derived BMECs [36]. Furthermore, iPSC-derived brain-like pericytes could be incorporated with neurons and astrocytes to further elevate BMEC TEER [36]. Here we investigate whether retinoic acid stimulation can synergize with this multicellular co-culture to further enhance the BBB properties of iPSC-derived BMECs. Such an isogenic human model would provide an important in vitro tool for helping to understand the human BBB in development, function, disease, and potentially identifying novel therapeutic approaches.

## Materials and methods

### iPSC differentiation to BMECs, Astrocytes, Neurons and Brain-like Pericytes

IMR90C4 (WiCell, Madison, WI) iPSCs were utilized for differentiations of all cell types. iPSCs were cultured between passages 30–50 on Matrigel (BD Biosciences, San Jose, CA) with daily mTESR1 (WiCell) medium changes. BMECs were differentiated as previously described [24]. In brief, iPSCs were singularized with Accutase (Life Technologies, Carlsbad, CA) and expanded to 30,000 cells/cm^2^ prior to the initiation of the differentiation. Unconditioned medium (UM; 100 mL Knock-out serum replacement, 5 mL non-essential amino acids (NEAA), 2.5 mL of gluta-max, 392.5 mL of Dulbecco’s modified Eagle’s medium (DMEM)/F12 (1:1) (Life Technologies) and 3.5 μL of β-mercapto-ethanol (Sigma, St Louis, MO) was exchanged daily for 6 days. UM was exchanged with EC +/+ medium (200 mL human Endothelial Serum-Free medium (hESFM; Life Technologies), 20 ng/mL basic fibroblast growth factor (bFGF; WiCell) and 1% platelet-derived bovine serum (PDS; Biomedical Technologies, Tewksbury, MA)) for 48 h supplemented with (treated) or without (non-treated) 10 μM retinoic acid (RA; Sigma). Neurons and astrocytes were derived from intermediary EZ-sphere and astro-sphere populations (respectively) as previously described [25]. EZ- and astro-spheres were singularized with Accutase and seeded at 25,000 cells/cm^2^ onto Matrigel coated plates. Neurons were differentiated and cultured in neuron medium [DMEM/F12 (70:30) supplemented with 1% penicillin–streptomycin, 2% B27 minus Vitamin A (Life Technologies) and 2 μg/mL heparin (Sigma)] with every-other day medium exchanges for 2 weeks. Astrocytes were differentiated and cultured in astrocyte medium (DMEM/F12 (1:1) supplemented with 1% NEAA, 1% N2 supplement; Life Technologies, and 2 μg/mL heparin) with every-other day medium exchanges for 2 weeks. Pericytes were differentiated from iPSCs as previously described [36]. Pericytes were derived in a two-step process. Initially iPSCs were differentiated in E6 medium [DMEM/F12, l-ascorbic acid-2-phosphate magnesium (Sigma), sodium selenium (Sigma), insulin (Sigma), NaHCO_3_ (Sigma), transferrin (Sigma)] supplemented with dorsomorphin (Tocris, Bristol, United Kingdom), SB431542 (Tocris) CHIR99021 (Tocris), bFGF (Waisman Biomanufacturing, Madison, WI), and heparin (Sigma)) to a neural-crest stem cell population. CD271 positive cells were enriched with neural crest stem cell microbeads (Miltenyi, Bergisch Gladbach, Germany) and seeded at 10,000 cells/cm^2^ onto non-coated plates (Corning). Following 6 days of expansion in E6 medium supplemented with 10% FBS (Thermofisher), pericytes were seeded at 10,000 cells/cm^2^ for co-culture studies with BMECs.

### Culture of 3T3 fibroblasts

3T3 mouse fibroblasts (ATCC, Manassas, VA) were cultured on Matrigel coated-plates with daily medium changes of DMEM (Life Technologies) supplemented with 10% FBS (Life Technologies). Mouse 3T3 fibroblasts served as a non-neural co-culture control.

### Initiation of co-culture experiments

BMECs were differentiated from iPSCs as previously discussed. Following differentiation, BMECs were singularized with Accutase and seeded onto Transwells (Corning Transwell polyester filters, 0.4 μm pore diameter; 1.12 cm^2^, Corning, NY) coated with collagen IV/fibronectin (Sigma) at a density of 1 × 10^6^ cells/cm^2^. Immediately following seeding, BMEC-coated Transwells were then placed into plates with either no cell types (monoculture), neuron: astrocyte (1:3) mixture, 3T3 fibroblasts, pericytes, or pericytes for the initial 24 h followed by a mixture of neurons: astrocytes (1:3) for the duration of the experiment. All experimental groups were in EC +/+ medium (hESFM supplemented with 1% PDS and 20 ng/mL bFGF) with or without retinoic acid for the initial 24 h and switched to EC ± medium (hESFM supplemented with 1% PDS) for the remainder of the experiment. All experiments occurred immediately following 48 h in co-culture unless otherwise stated.

### Transcytosis/accumulation measurements

Dextran (Alexa-Fluor^®^ 488, 10 kDa, 10 μM; Sigma) was used to measure the amount of transcytotic activity in the BMECs. Transwells were removed from co-culture and replaced in EC ± medium in the basolateral chamber. Dextran, diluted in EC ± was added to the apical side of the transwells on a rotating platform at 37 °C or 4 °C for 2 h. To determine the rate of transcytosis, media was collected from the basolateral chamber and read on a fluorescent plate reader. BMECs were then rinsed twice in phosphate buffer solution (PBS; Sigma) and lysed with radioimmunoprecipitation assay (RIPA; Sigma). After trituration the lysate was collected and quantified using a fluorescent plate reader, this value is the Dextran accumulated at this time point. Both transcytosis and accumulation values were normalized to protein content as determined by a bicinchoninic acid assay (BCA; Sigma).

### Trans-endothelial electrical resistance (TEER) measurements

Barrier tightness was measured by the voltage difference from the movement of ions across the Transwell membrane. Epithelial Volt/Ohm Meter (EVOM) with STX2 electrodes (World Precision Instruments) was used to measure the TEER value in ohms starting at day 9 of differentiation and continuing up to a week after purification to create an extended barrier tightness profile. The values are normalized by subtracting the background (TEER from a blank well) and then multiplied by the surface area (1.12 cm^2^) of the transwell filter and reported as ohms × cm^2^.

### Immunocytochemistry and analysis of tight junctions

Cells were fixed with cold methanol (100%; Sigma) or 2% paraformaldehyde (PFA; Sigma) in PBS for 15 min at room temperature. Following three washes in PBS the cells were blocked in 10% goat serum (Sigma) for 30 min. Cells were incubated with primary antibodies at 4 °C overnight on a rotating platform (Table 1). Following three PBS washes, cells were incubated with secondary antibodies for 1 h on a rotating platform at room temperature. Cells were incubated with a nuclear counterstain 4′,6-Diamidino-2-phenylindole dihydrochloride (DAPI; Sigma) for 15 min then washed in PBS and viewed on the Olympus epifluorescence microscope (Center Valley, PA). Images were analyzed using Image J, tight junction proteins (claudin-5, occludin and zo-1) were analyzed using threshold analysis and perimeter counting tools to determine the area of each image that exhibited occludin, claudin-5, or zo-1 immunoreactivity to determine area fraction index as previously discussed [25].

**Table 1:** Antibodies used for immunocytochemistry

Target antigen	Antibody species	Vendor	Clone/product number	Dilution
Pecam-1	Rabbit	Thermo Scientific	RB-10333	1:25
Glut-1	Mouse	Thermo Scientific	SPM498	1:500
Claudin-5	Mouse	Life Technologies	4C3C2	1:200
ZO-1	Mouse	Thermo Scientific	1A12	1:100
VE-Cadherin	Mouse	Santa Cruz	F-8	1:100
Occludin	Mouse	Life Technologies	OC-3F10	1:50
P-glycoprotein	Mouse	Thermo Scientific	P170(F4)	1:25
β-tubulin III	Rabbit	Sigma	T3952	1:500
GFAP	Rabbit	Dako	ZO334	1:500
NG2	Mouse	Millipore	MAB2029	1:100
PDGFR-β	Rabbit	Cell Signaling Technology	28E1	1:100

### P-glycoprotein efflux transporter assay

BMECs were removed from co-culture groups and placed in EC ± . Cyclosporin A (CsA, 10 μM; Sigma) was used as an inhibitor to P-glycoprotein efflux transporter (PGP), whereas the small molecule Rhodamine123 (Rh123, 10 μM; Sigma) is a PGP substrate. Half the wells of each experimental group were treated with CsA for 30 min in both apical and basolateral chambers of the transwell. A solution of diluted Rh123 in EC ± with and without CsA, was added to the apical side of all transwells for 1 h at 37 °C on a rotating platform. Media collected from the basolateral chamber is quantified on the fluorescent plate reader. To ensure that barrier integrity was maintained for the duration of the experiment, TEER values were monitored throughout the experimental timeline. NVU co-culture did not affect baseline Rh123 transport therefore the data is presented as a percentage change from no CsA treatment with each respective co-culture condition.

### Statistical analysis

**Fig. 1:**
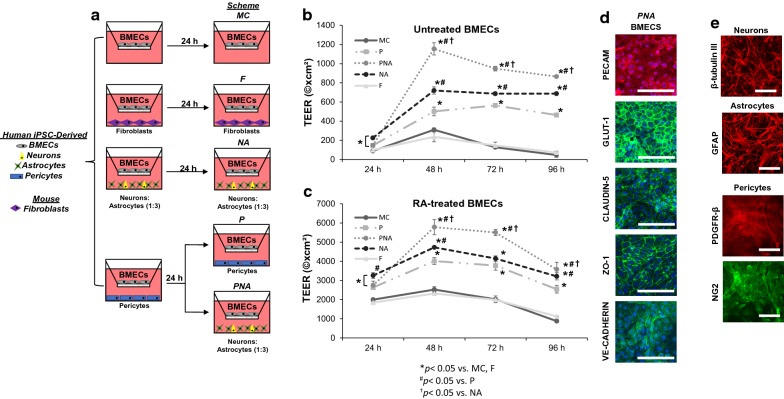
Induction of barrier properties brain microvascular endothelial cells (BMECs) following co-culture with pericytes, neurons, and/or astrocytes. **a** A completely isogenic blood brain barrier model was obtained by differentiating BMECs, neurons, astrocytes and pericytes from IMR90C4 induced pluripotent stem cells (iPSCs). BMECs cultured in monoculture conditions (MC) were directly compared to BMECs in co-culture up to 96 h past initial seeding onto Transwell filters. BMECs were co-cultured with mouse 3T3 fibroblasts (F), a mixture of iPSC-derived neurons and astrocytes (1:3; NA) or Pericytes (P). BMECs were initially co-cultured with pericytes for 24 h and then transferred to co-culture with a mixture of neurons and astrocytes (1:3) for the remainder of the experiment (PNA). **b** Trans-endothelial electrical resistance (TEER) profile of the experimental groups defined in panel (a) over a 96 h period. Statistical significance calculated using ANOVA. *p < 0.05 versus MC, F, ^#^*p *< 0.05 versus P, ^†^*p *< 0.05 versus NA. Values are mean ± SD of three replicates from a single differentiation and experiments were repeated for two additional differentiations for verification of reported statistical trends. **c** TEER profile of retinoic acid- treated BMECs over a 96 h period. *p < 0.05 versus MC, F, ^#^*p *< 0.05 versus P, ^†^*p *< 0.05 versus NA. Values are mean ± SD of three replicates from a single differentiation and experiments were repeated for two additional differentiations for verification of reported statistical trends. **d** Immunostaining of iPSC-derived BMECs following co-culture for 48 h in the PNA experimental condition probing for tight junction proteins, claudin-5 and ZO-1, endothelial cell markers, PECAM and VE-Cadherin, and glucose transporter, GLUT1. Scale bar = 100 μm. **e** Immunostaining of iPSC-derived neurons, astrocytes, and pericytes following co-culture with RA-treated BMECs for 48 h probing for β-tubulin III, GFAP, PDGFR-β, and NG2. Scale Bar = 100 μm

**Fig. 2:**
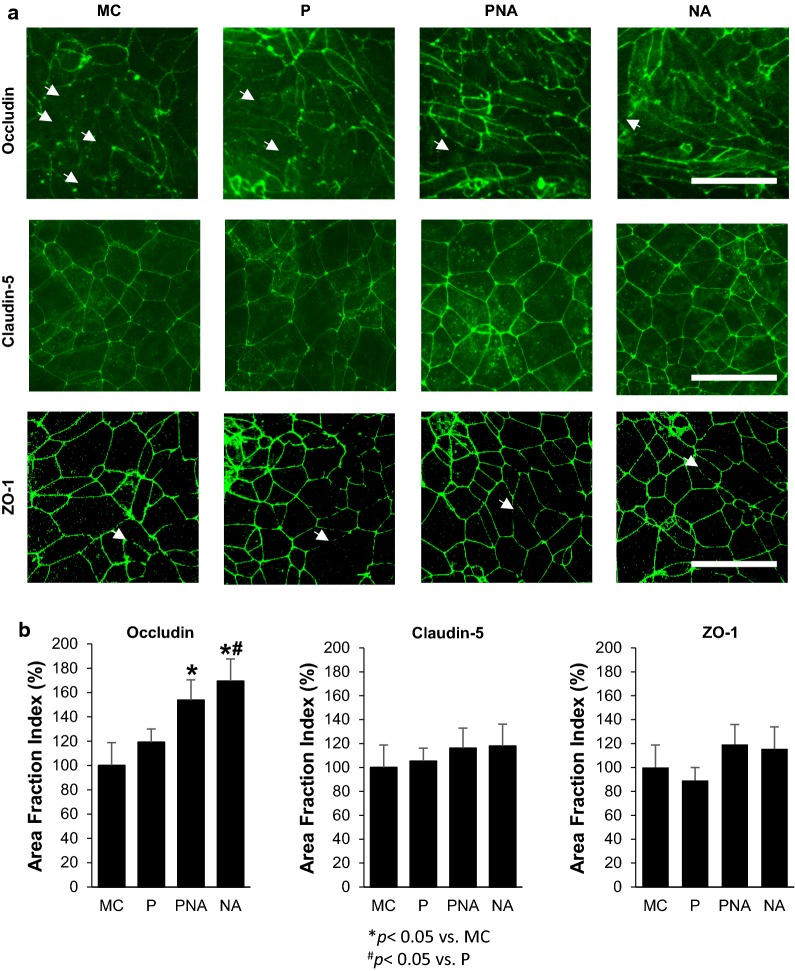
Analysis of tight junction continuity following co-culture. Localization of tight junction proteins, occludin, claudin-5, and ZO-1 were investigated in RA treated iPSC-derived BMECs following 48 h of co-culture with IMR90-iPSC-derived pericytes (P), neurons and astrocytes (1:3; NA) or pericytes (initial 24 h) followed by 24 h of neurons and astrocytes (1:3; PNA). **a** Immunocytochemistry of occludin revealed numerous discontinuous junctions (white arrows) in BMECs in monoculture compared to co-culture. Scale bar = 100 μm. **b** Quantification of the tight junction localization in BMECs in monoculture and co-culture conditions were calculated by the area of each image having immune-reactive occludin, claudin-5, or ZO1, resulting in area fraction index. Statistical significance calculated using ANOVA. Values are mean ± SD of three independent differentiations. *p < 0.05 versus MC, ^#^*p *< 0.05 versus P, and ^†^*p *< 0.05 versus the NA group

**Fig. 3:**
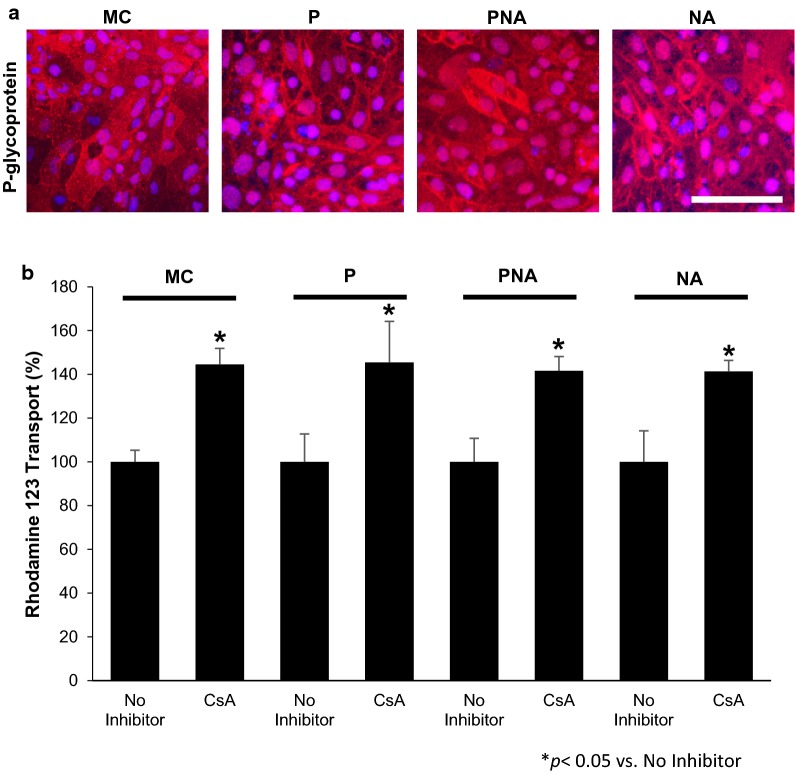
Evaluation of P-glycoprotein (PGP) efflux transporter expression and activity in RA-treated (BMECs). The expression and activity of PGP efflux transporters in BMECs in co-culture with iPSC-derived pericytes (P), neurons and astrocytes (NA), or pericytes, neurons, and astrocytes (PNA) were evaluated. **a** Immunolabeling of PGP efflux transporter following 48 h co-culture. **b** Activity of PGP efflux transporters in BMECs following 48 h of co-culture was assessed by the transport of the PGP substrate Rhodamine 123 with and without the PGP inhibitor cyclosporine A (CsA) from the apical to the basolateral chamber in the two-compartment co-culture model. Data is reported as percentage change from no-CsA treatment within each respective co-culture condition. Statistical significance was determined using a Student’s *t*-test. *p < 0.05 versus no CsA inhibition. One-way ANOVA determined there were no significant changes between each experimental group (co-culture conditions). *p *> 0.05 n.s.) Values are mean ± SD of three independent differentiations

**Fig. 4:**
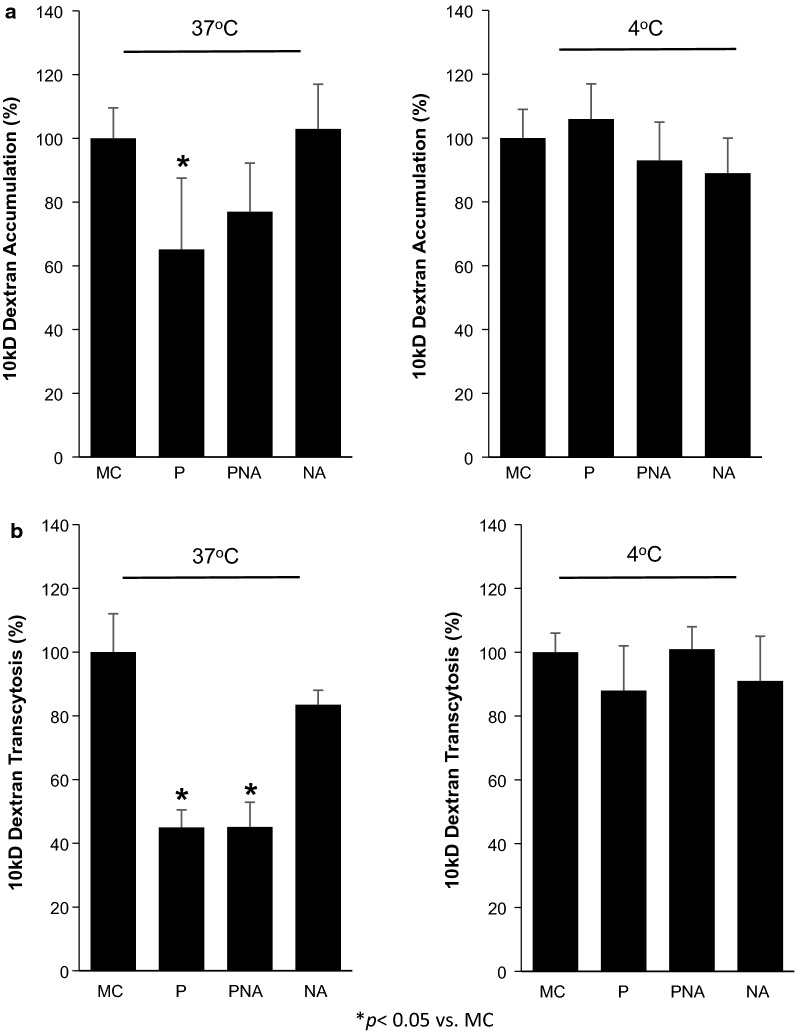
Determination of a fluorescently labeled dextran ability to cross RA-treated induced pluripotent stem cell (iPSC)-derived brain microvascular endothelial cells (BMECs) from the apical to the basolateral chamber in the two-compartment co-culture model. BMECs in monoculture (MC) or in co-culture for 48 h with either pericytes (P), neurons and astrocytes (NA), or pericytes, neurons, and astrocytes (PNA) were presented with a fluorescently tagged dextran for 2 h. Fluorescently-tagged dextran was measured within the BMEC population (**a**; accumulation) and from the bottom chamber (**b**; transcytosis) at both 37 °C and 4 °C. Raw fluorescence units are normalized to monoculture BMECs. Statistical significance was determined with ANOVA. Values are mean ± SD of three independent differentiations. *p < 0.05 versus MC

Data for Figs. 2, 3, 4 are presented as mean ± SD of three independent differentiations. Due to variable TEER baseline recordings, TEER values presented in Fig. 1 are mean ± SD of three replicates from a single differentiation. Two additional independent differentiations were repeated for verification of reported statistical trends. SigmaStat 3.0 software (Systat Software, San Jose, CA) was used for statistical analysis. Student’s t-test and one-way analysis of variance (ANOVA) with Holm-Sidak correction for all comparisons were utilized when appropriate and described within the figure legends for each particular data set.

## Results

### Co-culture of BMECs with Pericytes, Astrocytes and Neurons

Established protocols were used to differentiate BMECs [24], astrocytes (from EZ spheres) [25], neurons (from EZ-spheres) [25, 37], and brain-like pericytes (from neural crest progenitors) [36] from the IMR90C4 iPSC line. Following differentiation, iPSC-derived BMECs were seeded onto Transwells with different combinations of fully differentiated astrocytes, pericytes and neurons. The co-cultured cells were differentiated and maintained in their respective media prior to co-culture with BMECs. Immediately following seeding onto Transwells, BMECs were co-cultured with either iPSC-derived pericytes alone (P), a neuron and astrocyte mixture (1:3 NA) or a sequential pericyte co-culture followed by a neuron and astrocyte co-culture (PNA) (Fig. 1a). Monocultured BMECs (MC) and 3T3 fibroblast co-cultures (F) were used as comparative controls (Fig. 1a). All co-culture conditions were conducted initially in EC +/+ medium (hESFM supplemented with bFGF and PDS) for 24 h and then switched to an EC ± medium (hESFM supplemented with PDS) for the duration of the time course. To determine the effects that co-cultured cell types had on BMEC barrier properties, transendothelial electrical resistance (TEER) was monitored from 24 h after initiation of co-culture to 96 h (Fig. 1b). TEER values reached a maximum value at 48–72 h in all experimental conditions. Monocultured BMECs exhibited a maximum TEER value of 310 ± 19 Ω × cm^2^, whereas BMECs co-cultured with pericytes reached a maximum TEER value of 564 ± 21 Ω × cm^2^ (*p *< 0.05) and remained significantly elevated above monoculture and fibroblast co-culture for the duration of the experiment (*p *< 0.05). As previously demonstrated, EZ-sphere derived neurons and astrocytes (1:3) significantly elevated barrier tightening in BMECs with a maximum TEER of 720 ± 38 Ω × cm^2^ (*p *< 0.05), and remained significantly elevated (*p *< 0.05). Sequential BMEC co-culture with pericytes followed by a mixture of neurons and astrocytes resulted in the greatest induction of TEER above all other experimental groups at 48 h (1155 ± 64 Ω × cm^2^; *p *< 0.05) and remained significantly elevated above all co-culture groups (*p *< 0.05). All NVU co-culture groups elevated BMEC barrier tightness above monoculture and 3T3 fibroblast co-culture at all the time points of the experiment (*p *< 0.05). Finally, mouse 3T3 fibroblasts were utilized as a non-inductive co-culture cell type and did not elevate TEER above monoculture TEER levels at any time point (Fig. 1b, *p *> 0.05).

We have previously demonstrated that retinoic acid (RA) can significantly elevate the barrier properties of iPSC-derived BMECs [23]. To determine the potential synergistic effects of co-culture on a BMEC population that was exposed to RA during differentiation, we monitored TEER of RA-treated BMECs in all of the previously mentioned NVU co-culture groups (Fig. 1c). Similar to the untreated BMECs, we observed peak TEER values 48 h following seeding onto Transwells and the initiation of co-culture. Monocultured, RA-treated BMECs (M) had a maximum TEER value of 2525 ± 149 Ω × cm^2^ and were undistinguishable from BMECs in co-culture with 3T3 fibroblasts (F) 2321 ± 92 Ω × cm^2^ (*p *> 0.05). Sequential pericyte co-culture followed by neuron and astrocyte co-culture (PNA) significantly elevated TEER (5486 ± 396 Ω × cm^2^; *p *< 0.05) compared with pericyte co-culture (P) (4015 ± 173 Ω × cm^2^; *p *< 0.05) or neuron and astrocyte co-culture (NA) (4726 ± 55 Ω × cm^2^; *p *< 0.05). Similar to untreated BMECs, NVU co-culture elevated BMEC barrier tightness at all time points throughout the experiment (*p *< 0.05). To confirm that BMECs, neurons, astrocytes, and pericytes still expressed key markers after co-culture, we verified that RA-treated BMECs expressed the endothelial cell markers PECAM-1 and VE-cadherin, the BBB glucose transporter Glut-1, and tight junction proteins: claudin-5 and ZO-1 (Fig. 1d). Neurons expressed β-tubulin III, astrocytes expressed glial fibrillary acidic protein (GFAP), and pericytes expressed platelet derived growth factor receptor-β (PDGFR-β) and neuron glial antigen-2 (NG2) (Fig. 1e). Taken together, the PNA configuration provided the most significant induction of barrier properties and increased the TEER in RA-treated and untreated BMECs.

### PNA co-culture increases occludin localization in BMECs

Immunocytochemistry was utilized to investigate changes in tight junction protein localization during BMEC barrier tightening induced by NVU cell co-culture. Monoculture, RA-treated BMECs expressed junctionally localized occludin, claudin-5 and ZO-1 (Fig. 2a). Compared with monocultured RA-treated BMECs, co-culture with pericytes, a neuron: astrocyte mixture or pericytes followed by a neuron: astrocyte mixture, qualitatively increased occludin localization to the tight junctions (Fig. 2a). Quantification of area fraction index revealed that after 48 h of pericyte co-culture (P), BMECs had a slight, but statistically insignificant elevation in junctional occludin immuno-reactivity (19 ± 11%; *p *> 0.05) (Fig. 2b). However, BMECs in co-culture with pericytes for 24 h followed by a neuron: astrocyte (1:3) mixture for 24 h (PNA), exhibited significantly elevated junctional occludin levels (54 ± 17%; *p *< 0.05); however, the most significant boost in junctional occludin occurred after BMECs in co-culture for 48 h with a neuron: astrocyte (1:3) mixture (69 ± 17%; *p *< 0.05). However, the area fraction index of claudin-5 and ZO-1 were unchanged in BMECs following co-culture with pericytes, neurons, and astrocytes (Fig. 2b). These results suggest that enhanced barrier tightness following co-culture, at least in part, were the result of improved occludin localization and maintenance.

### P-glycoprotein efflux transporter activity is not affected by co-culture

To determine the effects of co-culture on P-glycoprotein (PGP) efflux transporter activity in RA-treated BMECs, the transport of rhodamine 123 across the BMEC monolayer was measured. Rhodamine 123 transport was compared between BMECs in monoculture and BMEC in co-culture with either pericytes (P), a neuron: astrocyte (1:3) mixture (NA), or pericytes followed by a neuron: astrocyte mixture (PNA). Immunocytochemistry revealed that, qualitatively, BMECs either in monoculture or in co-culture expressed similar levels of PGP (Fig. 3a). Correlating with the immunocytochemistry images, PGP function was also unchanged by any co-culture configuration. BMECs in monoculture exhibited an increase in rhodamine 123 following CsA inhibition (45 ± 7%; *p *< 0.05), benchmarking the baseline PGP activity in RA-treated BMECs (Fig. 3b). A similar increase in rhodamine 123 following CsA inhibition was observed in BMECs following co-culture (P: 45 ± 19%; PNA: 42 ± 7%; NA: 41 ± 5%; *p *< 0.05); however, these data were indistinguishable between BMECs in monoculture and co-culture (*p *> 0.05 n.s.). These data reveal that PGP is present and active in RA-treated BMECs as previously demonstrated [23] and co-culture with pericytes, neurons and astrocytes alone or in combination does not affect this activity.

### Non-specific transcytosis in RA-treated BMECs is decreased in pericyte co-cultures

In previous studies, pericytes have been demonstrated to reduce the non-specific transcytosis of tracers and large molecules through BMECs [6, 38, 39]. To determine if co-culture with brain-like pericytes could also elicit similar effects in iPSC-derived RA-treated BMECs, the effects of co-culture on uptake and transcytosis of 10 kDa fluorescently tagged dextran was employed. BMECs in monoculture or 48 h following co-culture with pericytes (P), a neuron: astrocyte (1:3) mixture (NA), or pericytes followed by a neuron: astrocyte mixture (PNA) were incubated with fluorescently-tagged dextran in a Transwell setting. After addition of dextran to the top chamber, transport was measured both as cellular accumulation in BMECs and by accumulation in the lower chamber (transcytosis) then normalized to accumulation and transcytosis using monocultured BMECs (100%) (Fig. 4a, b). Following pericyte co-culture, BMECs exhibited significantly lower accumulation and transcytosis of dextran (65 ± 22% and 45 ± 5%; respectively; *p *< 0.05). While pericyte co-culture followed by neuron: astrocyte co-culture (PNA) did not yield a statistically significant decrease in dextran accumulation (77 ± 15%; p > 0.05), the amount of dextran that transcytosed across BMECs was significantly reduced (45 ± 8%; *p *< 0.05). Neuron: astrocyte co-culture (NA) lacking pericyte influences did not significantly affect dextran accumulation or transcytosis across BMECs (103 ± 14%, 83 ± 5%; respectively; *p *> 0.05). Non-specific transcytosis is minimal in BMECs compared to peripheral endothelial cells, similarly less than 0.05% of the dextran added to the apical chamber accumulated within the cell and less than 0.04% crossed the BMEC monolayer. To rule out the changes in transcytosis being a result of the increased passive barrier properties upon co-culture, dextran accumulation and transcytosis was also conducted at 4 °C conditions where vesicular transport processes are significantly inhibited (< 0.003% of the total dextran), but passive diffusion is comparatively unaffected (Fig. 4a, b). Under these conditions, the dextran transport was unaffected following pericyte co-culture compared to monoculture conditions, indicating that the increases in TEER do not explain the change in dextran transport. Taken together, these data indicate that brain-like pericyte co-culture alone or in conjunction with neurons and astrocytes can decrease the uptake and non-specific transcytosis of large molecules across RA-treated BMECs.

## Discussion

This study demonstrates that human iPSCs can be utilized to derive BMECs, neurons, astrocytes, and brain-like pericytes that can be combined to generate an enhanced in vitro NVU model with elevated barrier and reduced nonspecific transcytosis properties. The unique advantage of the model presented here is that each of the cell types is derived from the same human iPSC source, enabling a host of applications from modeling human disease to personalized medicine. A major limitation of other NVU models is that one or more of the cell types is from a different source, either neonatal human or animal, impeding translational aspects [14, 32, 40–42]. However, iPSC technology allows for each cell type of the model to be generated independently under ideal conditions prior to co-culture [24, 36, 37, 43, 44]. Several studies have shown that BMECs can be successfully differentiated from iPSCs and they express a wide array of critical barrier properties including active nutrient and efflux transporters [11, 21, 24, 27, 33]. Previously, it has been demonstrated that astrocytes and neurons derived from iPSCs via an intermediary EZ-sphere/astro-sphere population, can enhance BMEC properties such as increased TEER, reduced para-cellular permeability, and enhanced junctional localization of occludin and claudin-5 [25, 33]. The greatest barrier tightening in BMECs was observed following coculture with a mixture of neurons and astrocytes (1:3), closely mimicking the composition of neurons and astrocytes in the human brain [45, 46]. Formerly, the limiting component of the stem cell-derived BBB model was the unavailability of iPSC-derived pericytes having brain-specific attributes [35, 47, 48]. The recent ability to derive brain-like pericytes from iPSCs is invaluable to modeling the NVU because pericytes enhance several critical BBB properties. While we have reported the elevation of TEER in the baseline PNA model [36], the contribution of brain-like pericytes to the isogenic BBB model in terms of non-specific transctytosis and efflux transporter function had not yet been evaluated. Moreover, the impact of combining enhanced RA-treated BMECs, with iPSC-derived brain-like pericytes, neurons, and astrocytes was unknown.

We observed the greatest barrier induction when BMECs were first co-cultured with pericytes for 24 h then co-cultured with a mixture of neurons: astrocytes (1:3). Our laboratory previously demonstrated that primary human brain pericytes and primary human neural progenitor cell-derived neurons and astrocytes also enhanced barrier properties in iPSC-derived BMECs [23]. In BBB development, BMECs are first subject to cues from pericytes and then later are influenced by astrocytes, motivating the sequential co-culture utilized here [5–7]. To further enhance the barrier tightening capabilities of BMECs, we manipulated retinoic acid signaling, a vital developmental pathway that has been implicated in the induction of brain-specific properties during development while also influencing brain and anterior spinal cord development [49–52]. Previously, it has been demonstrated that RA increased barrier function and junctional protein localization in iPSC-derived BMECs [23, 53]. Here we show that RA-treated BMECs further respond to co-culture with iPSC-derived brain-like pericytes, neurons, and astrocytes to elevate TEER to near in vivo levels (~ 2000–6000 Ω × cm^2^) [54]. Elevations in TEER are often associated with changes in tight junction protein levels or localization [41, 54–57]. Even in BMEC models where sufficient tight junctions are present, and their protein levels remain unchanged by a particular stimulus, the junctional continuity can be enhanced and correlate with increased TEER [23, 25]. Similar to these studies, we observed an increase in the junctional continuity of occludin following co-culture suggesting a potential role of occludin organization in increasing TEER levels. Interestingly, the greatest increase in occludin junctional continuity was observed following neuron: astrocyte (1:3) co-culture and did not correspond to the greatest increase in TEER amongst experimental groups. Pericyte co-culture alone did not yield a statistically significant change in junctional localization of occludin despite a qualitative improvement in junctional localization, suggesting that the corresponding increase in TEER observed in pericyte co-culture may be associated with mechanisms other than occludin localization.

Another vital property of the BBB is the presence and activity of efflux transporters. Previous studies have indicated that co-culture conditions, primarily with astrocytes, can enhance PGP protein expression and activity levels in primary and immortalized BMECs [8, 58, 59]. iPSC-derived BMECs under baseline conditions express functional PGP [22–25, 43]. In this study, we demonstrated that regardless of co-culture conditions, BMECs expressed active efflux PGP transporters that were unaffected by co-culture. Similar to these observations, previous studies have demonstrated that co-culture did not alter PGP expression or activity [25, 60–62]. The final critical BBB component that was investigated is that of a significantly reduced rate of non-specific transcytosis in BMECs compared to endothelial cells of the periphery [6]. Following iPSC-derived brain-like pericyte co-culture, we observed a significant reduction in uptake and transport of a large dextran molecule in iPSC-derived BMECs, reprising a similar role for pericytes in BBB development and regulation [6, 38].We utilized a transwell co-culture system to evaluate the effects that astrocytes, neurons, and pericytes have on BMEC barrier phenotypes. A number of effectors secreted by NVU cell types have been previously identified to enhance barrier properties in non-stem cell BBB models. Specifically, pericytes induced claudin-5 expression and enhanced tight junction localization via platelet-derived growth factor and transforming growth factor beta-1 signaling [6, 8]. Astrocyte-induced effectors: sonic hedgehog, angiopoietin 1/2, and apolipoprotein E upregulate, alter the subcellular distribution, and modify tight junction proteins [63–65]. The effectors involved in the human BBB are relatively unknown, and further studies will be needed to examine whether previously identified factors or possibly new human-specific NVU secreted factors are acting in the model. The Trans-well system enabled several barrier properties to be monitored simultaneously while maintaining flexibility in altering co-culture schemes. Additional co-culture architectures such as engineered vessels [28, 29], microfluidic channels [66], and spheroid models can be utilized to further mimic the in vivo barrier [67].

## Conclusions

Here we demonstrate a human isogenic BBB model comprised of BMECs, neurons, astrocytes and brain-like pericytes all derived from the same iPSC line. The model performed similarly to other non-isogenic BBB models with expression of several key BMEC proteins and enhanced tight junction continuity while also displaying near in vivo TEER levels and significantly reduced transcytosis rates. Importantly, an entirely isogenic human BBB model will likely enable personalized medicine approaches with potential applications in tissue engineering, disease modeling and pharmaceutical screening.

## Data Availability

The datasets used and/or analysed during the current study are available from the corresponding author on reasonable request.

## Abbreviations

BBB: blood–brain barrier
BMECs: brain microvascular endothelial cells
iPSCs: induced pluripotent stem cells
NVU: neurovascular unit
RA: retinoic acid
PGP: p-glycoprotein
TEER: trans-endothelial electrical resistance
