# Correction to: An isogenic neurovascular unit model comprised of human induced pluripotent stem cell-derived brain microvascular endothelial cells, pericytes, astrocytes, and neurons

**DOI:** 10.1186/s12987-019-0151-8

**Published:** 2019-09-10

**Authors:** Scott G. Canfield, Matthew J. Stebbins, Madeline G. Faubion, Benjamin D. Gastfriend, Sean P. Palecek, Eric V. Shusta

**Affiliations:** 1 Department of Chemical and Biological Engineering, University of Wisconsin, Madison, WI 53706 USA; 2 Present Address: Department of Cellular and Integrative Physiology, Indiana University School of Medicine, 620 Chestnut Street, Terre Haute, IN 47809 USA

## Correction to: Fluids Barriers CNS (2019) 16:25 10.1186/s12987-019-0145-6

Following publication of the original article [1], the author has reported that in Figure 1 (b and c) the y-axis TEER (© × cm^2^) should be replaced with TEER (Ω × cm^2^).

It has been corrected in the original article as well.

The publisher apologizes for any inconvenience caused by this error.
